# Within-person Relations Between Social Skills and Symptoms of Oppositional Defiant Disorder and Conduct Disorder from Preschool to Adolescence – A Birth Cohort Study

**DOI:** 10.1007/s10802-025-01298-x

**Published:** 2025-02-18

**Authors:** Silje Merethe Husby, Lourdes Ezpeleta Ascaco, Lars Wichstrøm

**Affiliations:** 1 Department of Psychology, Norwegian University of Science and Technology, 7491 Trondheim, Norway; 2 Department of Child and Adolescent Mental Health, St. Olav Hospital, Trondheim, Norway; 3 Unitat d’Epidemiologia I de Diagnòstic en Psicopatologia del Desenvolupament, Barcelona, Spain; 4 Departament de Psicologia Clínica I de La Salut. Edifici B, Universitat Autònoma de Barcelona, Barcelona, Bellaterra Spain

**Keywords:** Oppositional defiant disorder, Conduct disorder, Social skills, Social competence, RI-CLPM

## Abstract

Effective treatment of oppositional defiant disorder (ODD) and conduct disorder (CD) is dependent on etiological knowledge about what factors contribute to their development and maintenance. Previous research points to a role for social skills in this process, but findings are mixed, possibly due to methodological factors. A sample drawn from two birth cohorts (*n* = 1,079, 50.1% girls) in the city of Trondheim, Norway, was assessed biennially from age 4 to 16 years. Social skills were rated by teachers and parents and measures of symptoms of ODD and CD were obtained through diagnostic interviews of parents and children (from age 8). Bidirectional relations between within-person changes in social skills and symptoms of ODD and CD, respectively, were assessed by a random intercept cross-lagged model. Increased parent-reported social skills predicted reduced symptoms of both ODD and CD from preschool to adolescence, while teacher-rated social skills predicted reduced symptoms of ODD from 4 to 14 years but predicted reduced symptoms of CD from 6 to 8 years only. Symptoms of ODD and CD predicted reduced teacher-rated social skills on two occasions (6 to 8 years for CD and 8 to 10 years for ODD), but there were no reciprocal paths involving parent-rated social skills. Findings provide support for including social skills training in the prevention and treatment of symptoms of ODD and CD.

**Supplementary Information:**

The online version contains supplementary material available at 10.1007/s10802-025-01298-x.

Oppositional defiant disorder (ODD) and conduct disorder (CD) are prevalent in childhood and adolescence (3.2% and 3.3%, respectively; Canino et al., 2010) and are among the predominant reasons for referral to child- and adolescent psychiatry (Wichstrøm et al., 2014). They are associated with significant impairment and risk for long-term difficulties (Loeber et al., 2000) and considerable societal costs (Boldrini et al., 2022). Although several treatment programs appear effective (Riise et al., 2021), findings are mixed (Hendriks et al., 2018), and meta-analyses point to a lack of evidence for long-term effects (Boldrini et al., 2022), highlighting a need for improving therapeutic and preventive efforts. We argue that such work should be based on etiological knowledge. It is widely assumed that impaired social skills may contribute to the development of behavioral disorders (Huber et al., 2019), but several measurement- and analytical issues which will be detailed below limit what conclusions can be drawn.

## Reciprocal Relation Between Social Skills and Behavioral Disorders

Social competence broadly refers to the ability to effectively manage social relations to meet short and long-term goals (Rose-Krasnor, 1997) and includes age-appropriate social skills like helping others, sharing, cooperating, being assertive, or responding appropriately in conflict and non-conflict situations. Social skills can thus be regarded as a behavioral component of social competence (Little et al., 2017). Children lacking in these skills are likely to experience little success and much conflict in their interactions, making them undesirable playmates and prone to rejection from peers. This could again lead to frustration and aggressive or acting-out behavior (Bornstein et al., 2010). Previous research has reported that deficits in social skills predict later behavior problems (e.g., Bornstein et al., 2010), although others have failed to find the same (e.g., Obradovic & Hipwell, 2010).

Considering the opposite direction of effects, children evincing aggressive, under-controlled, or antisocial behavior may be rejected or ignored by socially competent age-mates (e.g., Bierman, 2004), which further hampers their acquisition of and training in social skills (Bornstein et al., 2010). These children may seek out and affiliate with other aggressive children, who may model and reinforce less socially competent and more disruptive behavior (Patterson et al., 2000), possibly reflected in research chronicling predictive relations between behavior problems and later reductions in social skills (e.g., Obradovic & Hipwell, 2010). Indeed, some have found reciprocal relations between social skills and behavior problems (e.g., Memmott-Elison & Toseeb, 2023), whereas others have failed to find any predictive relations (Burt et al., 2008) or found that the relations are present at some ages but not others (e.g., Burt & Roisman, 2010). Given such inconsistent findings, it seems important to clarify any longitudinal relations.

Social skills training is included in several intervention programs, and meta-analyses document small to moderate effects on behavior problems, but there is little evidence for long-term effects (Beelmann & Lösel, 2021). While some intervention studies find that reductions in symptoms are, in part, mediated by enhanced social skills (e.g., Hektner et al., 2014), others fail to find the same (Katzmann et al., 2019). Thus, whether enhanced social skills truly forecast reduced symptoms of behavioral disorders, in the long run, needs to be examined directly. A task arguably best addressed in observational research.

## Methodological Issues

Several methodological limitations hinder the ability to draw etiological implications from current findings. These include measurement issues, common method and rater bias, and data-analytical approaches that limit causal interpretation.

### Rating Scales Versus Diagnostic Interviews

A vast majority of research on behavior problems has been carried out using checklists or rating scales completed by parents or teachers. Although valuable, parent and teacher reports may be subject to errors and biases (Bennett & Offord, 2001), reflected in findings showing that a group of high scorers on such questionnaires only contained 17–42% true positives of *diagnosed* behavioral disorders based on clinical interviews (Sveen et al., 2013). Such an arguably low sensitivity may be attributed to lacking or ambiguous onset, frequency, duration, and intensity criteria within these instruments, as well as the potentially limited experience of what is normative behavior or not among the respondent who is set to evaluate the behavior (e.g., parents). In addition, the often-used general measure of externalizing problems in the Child Behavior Checklist (CBCL; Achenbach, 1991) also contains items pertaining to impulsivity and difficulties in regulating attention and activity, which some argue may rather be symptoms of ADHD (Hay et al., 2010). Although ADHD is grouped with ODD and CD in the category of externalizing disorders it is, at its core, a neurodevelopmental disorder with a distinct genetic risk profile (Azeredo et al., 2018). Thus, the inclusion of symptoms that actually may be symptoms of ADHD could muddle the measurement of ODD and CD (Hay et al., 2010), and perhaps this could be one factor explaining the variability in previous findings. In contrast, we argue that structured clinical diagnostic interviews administered by trained interviewers provide a more valid measure of symptoms of behavioral disorders. To the best of our knowledge, this study is the first to employ diagnostic interviews to measure clinical symptoms of ODD and CD in relation to social skills.

### Separating ODD and CD

Theory and research, to an increasing degree, underscore the differences between ODD and CD with regard to predictors, covariates, and outcomes (Burke et al., 2022; Wiesner et al., 2015). Although there is some overlap with respect to core symptoms and features, they also include behaviors that are specific and exclusive to each one (Burke et al., 2024). Their response to treatment interventions has also been found to differ (e.g., Kolko et al., 2009). It is yet to be examined whether the developmental dynamics of ODD and CD are differentially related to social skills. Since social skills training figures in many prevention and treatment interventions, and treatments are targeted at the within-person level (Hamaker et al., 2015; Speyer et al., 2024), it would be of etiological and clinical interest to illuminate the specific within-person relations from social skills to ODD and CD respectively. Although one cross-sectional study found that certain aspects of social skills were putative correlates to both ODD and CD symptoms (Wiesner et al., 2015), we are not aware of any longitudinal studies examining this.

### Common Method and Rater Bias

With some exceptions (e.g., Bornstein et al., 2010; Burt et al., 2008), single-informant reports on both behavior problems and social competence have been the norm—possibly inflating associations due to single-informant biases and shared method variance (Huber et al., 2019). Furthermore, reporter effects may change during development (Merrell, 2001). This may be especially salient in terms of parental reports on social competence, as children and parents spend decreasing amounts of time together with age (Memmott-Elison & Toseeb, 2023), leaving parents with less access to their children’s social arenas and social behaviors (Renk & Phares, 2004). Teachers, however, may see children in a different social context, which arguably varies less with age. To limit common rater and context effects, we utilize reports from teachers, children, and parents.

### Analysis: Between-person Differences Versus Within-person Changes

Previous research has typically utilized regression-type or cross-lagged panel models (CLPM), which produce results that are a mixture of between and within-person variance (Berry & Willoughby, 2017; Hamaker et al., 2015). Because causality operates on the within-person level, between-person differences need to be filtered out so that the findings can better inform etiological inferences. We are aware of four studies that have applied a within-person approach to the present topic (Memmott-Elison & Toseeb, 2023; Speyer et al., 2024; Williams et al., 2024; Zondervan-Zwijnenburg et al., 2022). The results have been mixed. Memmott-Elison and Toseeb (2023) found reciprocal relations between prosocial behavior and behavior problems from 3 to 14 years (but from conduct problems to prosocial behavior only from 3 to 5 years), and Williams and colleagues (2024) found reciprocal negative relations between prosocial behavior and conduct problems from 4–15 years. Speyer and colleagues (2024), on the other hand, found that self-reported (but not teacher-reported) aggression at 11 years predicted decreased prosocial behavior at 13 years, but not from 13 to 15, and not the other way around; while Zondervan-Zwijnenburg and colleagues (2022) found some initial support for a negative within-person cross-lagged relation from behavior problems to later externalizing behavior in from 7 to 11 years, but concluded that overall there was little to no support for any predictive within-person relation between the two.

The abovementioned studies differ from the present in that they have all utilized rating scale measures of externalizing behavior problems or aggression, as opposed to diagnostic interviews geared towards symptoms of diagnosable disorders. Furthermore, they all focus on the concept of *prosocial behavio*r. Prosocial behavior is generally defined as voluntary helping, sharing, and supporting behaviors. Although a significant part of social skills, the latter encompasses a broader range of social behaviors, such as communication, cooperation, assertion, engagement, and self-control (Gresham et al., 2011; Little et al., 2017). Existing theories on behavior disorders suggests that these subskills, or lack thereof, are related to ODD and CD (e.g., Gilmour et al., 2004; Wiesner et al., 2015; Zhang et al., 2023). These additional subskills are also often targeted in some of the widely used psychosocial treatments of behavioral disorders, which involve training children in communication skills, emotional regulation, social information processing, and conflict resolution (e.g., Beelmann & Lösel, 2021; Goertz-Dorten et al., 2017; Webster-Stratton & Reid, 2018). Thus, examining the longitudinal within-person relations between changes in the broader concept of social skills and symptoms of ODD and CD separately could aid in providing a more precise description of how social skills are related to these two disorders across development. Such knowledge could aid in choosing and designing disorder-appropriate interventions. Furthermore, both Memmott-Elison and Toseeb (2023), Speyer et al. (2024), and Williams et al. (2024) measured both behavior problems and prosocial behavior using subscales from one questionnaire only, as well as using the same reporter on both prosocial behavior and behavior problems—issues that may have led to inflated prospective relations. With regards to Zondervan-Zwijnenburg et al. (2022), as the authors themselves profess, there is some uncertainty attached to the findings due to the sample size being below the recommended cut-off for use with random intercept cross-lagged panel models (RI-CLPM) (Masselink et al., 2018).

## Current Research

It is viable that poor social skills may be involved in the development and maintenance of symptoms of ODD and CD, but any causal inferences drawn from available research have been limited by factors pertaining to measurement, raters, and methods of analysis. We therefore test the hypothesis that reduced social skills will predict increased DSM-5-defined symptoms of ODD and CD, and vice versa, at the within-person level, using a multi-informant design and applying diagnostic interviews in a 12-year seven-wave study of a birth cohort sample.

## Methods

### Participants and Procedure

The Trondheim Early Secure Study (TESS) consists of children of the 2003 and 2004 birth cohorts in Trondheim, Norway (*N* = 3,456*)* (Steinsbekk & Wichstrøm, 2018). A letter of invitation, along with the Strengths and Difficulties Questionnaire (SDQ) 4–16 version (Goodman, 1997), was sent to their homes, and parents brought the completed SDQ to the routine 4-year health check-up (*n* = 3,358). Parents were informed about TESS using procedures approved by the Regional Committee for Medical and Health Research Ethics Mid-Norway, and written consent was obtained.

To increase statistical variance and thus power, we oversampled children with emotional or behavioral problems based on their SDQ total difficulties score and then divided the sample into four strata (cut-offs: 0–4, 5–8, 9–11, and 12–40). Using a random number generator, drawing probabilities to participate increased with increasing SDQ scores (0.37, 0.48, 0.70, and 0.89). Of the 1,250 children randomly drawn, 1,007 were enrolled at T1. The drop-out rate after consent did not differ according to SDQ score [(*t* = 0.17, *df* = 1, *p* = 0.86) or gender (χ^2^ = 1.02, *df* = 1, *p* = 0.31)]. The mean age at the first assessment was 4.7 years (SD = 0.30, 49.9% males). Retesting occurred at 6 years (T2): *M*_*age*_ = 6.7 years, *SD* = 0.25; 8 years (T3): *M*_*age*_ = 8.8, *SD* = 0.24; 10 years (T4): *M*_*age*_ = 10.5 years, *SD* = 0.16; 12 years (T5): *M*_*age*_ = 12.5, *SD* = 0.15;14 years (T6): *M*_*age*_ = 14.4 years, *SD* = 0.16, and 16 years (T7): *M*_*age*_ = 16.9, *SD* = 0.31. Overall, 1,079 participants had information from at least one wave of data collection and comprised the analytical sample.

The attrition rate was lower for those with higher teacher-rated social skills at the previous assessment point for age 6 (*OR* = 0.49, *p* = 0.003), age 8 (*OR* = 0.46, *p* = 0.017), and age 12 (*OR* = 0.29, *p* < 0.001). Even so, the combined effects of the predictors of attrition were small, varying between Cox and Snell’s Proxy *R*^*2*^ 0.017–0.023. Little’s MCAR test (Li, 2013) was χ^2^ = 5286.06, df = 4904, *p* < 0.001, supporting that data was not missing completely at random (MCAR). However, as the normed test was 1.08, data was likely missing at random (MAR).

### Measures

#### The Preschool Age Psychiatric Assessment (PAPA) and the Child and Adolescent Psychiatric Assessment (CAPA)

The PAPA is a semi-structured psychiatric interview with parents about their preschool-age children (Egger et al., 2006) and was administered at ages 4 and 6 (T1 and T2). In the DSM-5, ODD is comprised of eight symptoms, whereas CD has 15. Six CD symptoms that occur in the CAPA are deemed age-inappropriate for 4–6-year-olds, and are not included in the PAPA (e.g., “Stolen while confronting a victim”). Parents were asked to base their report on the preceding three months. Blinded raters re-coded 9% of the interview audio recordings. The interrater reliabilities using intra-class correlations (ICC) were ODD = 0.97 and CD = 0.91. At ages 8 to 14 years (T3-T6), we applied the child and adolescent version of the PAPA, the CAPA (Angold & Costello, 2000); this time, we also interviewed the children. Parents and children were interviewed separately. A symptom was regarded as present if it was reported by at least the child or the parent (Angold & Costello, 2000). The prevalence of a fully diagnosable behavior disorder, especially CD, will likely be low in a non-clinical sample (Husby & Wichstrøm, 2017), but sub-clinical levels of a disorder may be present and predictive of a later disorder at an early age even though the diagnostic threshold is not met until years later (Fergusson & Horwood, 1995; Keenan et al., 2011). Thus, in the case of a non-clinical sample starting in early childhood, a continuous approach may prevent a loss of information, compared to diagnostic cut-offs (Keenan et al., 2011; Rolon-Arroyo et al., 2014). Symptom counts were therefore used in the analyses. The interrater reliabilities from blinded recodings of 15% of the interviews were ICC = 0.90 for ODD and ICC = 0.85 for CD. The interviews were administered by experienced interviewers who were trained by the team responsible for developing the PAPA and the CAPA.

#### Schedule for Affective Disorders and Schizophrenia for School-Aged Children

At age 16 (T7), symptoms of ODD and CD were assessed with the semi-structured diagnostic interview Schedule for Affective Disorders and Schizophrenia for School-Aged Children – Present Life Version (K-SADS-PL DSM-5; Kaufman et al., 1997). A 3-month primary period was used. Trained interviewers conducted separate interviews with parents and adolescents, and a symptom was regarded as present if it was reported by either one of them. Interrater reliabilities from blinded recodings of 114 interviews were ICC = 0.92 for the number of ODD symptoms and ICC = 0.84 for CD symptoms.

PAPA, CAPA and K-SADS specifically question about ODD and CD symptoms as they occur in the DSM-IV/5, and they all contain similar interviewer instructions about probes and scoring criteria to ensure the symptom in question is present. They are regarded as among the evidence-based best-practice assessment methods for ODD and CD (Burke et al., 2024), making it reasonable to assume that they will converge in identifying the same children and youth to exhibit symptoms of ODD and CD.

#### The Social Skills Rating System and The Social Skills Improvement-Rating Scales

To assess social skills, we used the Social Skills Rating System (SSRS; Gresham & Elliot, 1990). At ages 4 and 6 years (T1 and T2), the preschool version was applied, whereas at ages 8 and 10 years (T3 and T4), the elementary version was used, and from ages 12 to 16 (T5 to T7) we applied the revised version, the Social Skills Improvement System-Rating Scales (SSIS-RS; Gresham & Elliott, 2008). The SSRS is a broad, multi-rater assessment of children’s social skills. In this study, we utilized the parent (α = 0.83–0.93) and teacher (α = 0.93–0.95) versions. A 4-point Likert scale is applied with higher scores indicating better skills, and a mean score of the answers to the four subscales cooperation, assertiveness, self-control, and responsibility (the latter is found in the parent form only) were used. The SSIS-RS assesses social skills on three additional subscales, but to ensure the stability of measurement across time we utilized only the same subscales as for the SSRS (teacher version α = 0.90–0.94, parent version α = 0.90–0.96). In senior high school (age 16) teachers were considered not to be valid reporters of children’s social skills. At that age, adolescents mostly have different teachers for different subjects in school, at least in the Norwegian schooling system, and thus each teacher possesses limited knowledge about a specific student. Thus, only the parents were asked to respond to the SSIS-RS.

As mentioned, previous RI-CLPM research on this topic has mainly focused on prosociality. In an attempt to gauge the extent to which the current findings involving the broader construct of social skills would generalize to the narrower construct of prosociality, we estimated the correlation between the subscale allegedly closest to prosociality, namely Cooperation, and the other scales. Correlations for parent-ratings ranged from 0.80—0.92, whereas the correlations for teacher ratings ranged from 0.87—0.95. We therefore expect findings to generalize to Cooperation as well.

### Analysis Plan

Analyses were conducted using structural equation modeling (SEM) in Mplus 8.5, employing a robust maximum likelihood estimator. Missingness was handled with a full information maximum likelihood (FIML) procedure under the assumption that the data was MAR. To arrive at correct population estimates due to the stratification, data were weighted with a factor corresponding to the number of children in the stratum divided by the number of participating children in that stratum. As the number of ODD and CD symptoms was expected to be heavily right-skewed, we used the natural log.

The RI-CLPM allows for the separation of variance into a stable between-person part (consisting of three latent random intercepts loading on social skills, symptoms of ODD, and symptoms of CD) and a within-person part, which estimates changes from one’s own mean level in a variable (e.g., social skills) as a function of changes in that variable at the previous measurement point (autoregression) and in the predictor (i.e., ODD or CD symptoms; a cross-lagged effect). Parent-rated and teacher-rated social skills were analyzed in separate models.

To examine whether cross-lagged paths differed between ages, a model where the cross-lagged paths in question were allowed to vary across time was compared to a model where paths were set to be identical using the Satorra-Bentler scaled Chi-square test (Bryant & Satorra, 2012). As we conducted a high number of comparisons, we adjusted for the false discovery rate (Benjamini & Hochberg, 2000). To ease comparison with prior studies using a blend of within- and between-person information, the analyses were also run with an ordinary CLPM.

## Results

The means and standard deviations of all study variables are presented in Table S1 (available online). The number of ODD symptoms increased towards a peak at age 8 followed by a small gradual decline. At age 16, the number of ODD symptoms dropped substantially. The number of CD symptoms remained reasonably stable towards age 16 when a marked increase occurred. As expected, correlations between teacher and parent reports on social skills were weak to moderate (between 0.12– 0.34). The same was true for the correlations between teacher-rated social skills and symptoms of ODD (−0.10 – −0.35) and CD (−0.10 – −0.28), and parent rated-social skills and symptom of ODD (−0.11 – −0.35) and CD (−0.09 – −0.28) (Table S2).

### Model Fitting

The RI-CLPM involving parent-rated social skills revealed a somewhat sub-optimal fit to the data, χ^2^ = 304.94, df = 129, *p* < 0.001, Comparative Fit Index (CFI) = 0.937, Tucker-Lewis Index (TLI) = 0.897, Root-mean-square error of approximation (RMSEA) = 0.036, 90% CI: 0.030–0.041. However, a model with 4-year autoregressive lags included did fit the data adequately, χ^2^ = 204.30, df = 114, *p* < 0.001, CFI = 0.968, TLI = 0.940, RMSEA = 0.027, 90% CI: 0.021–0.033.

**Fig. 1:**
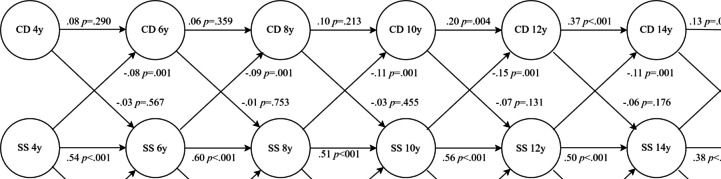
RI-CLPM showing relationships between symptoms of ODD, symptoms of CD, and parent-reported social skills. *Note*. SS = social skills—parent-reported; ODD = symptoms of ODD, CD = symptoms of CD. Standardized path coefficients

Setting all the paths from social skills to ODD and to CD at ages 4–16 to be similar did deteriorate model fit slightly, Δχ^2^ = 18.45, *df* = 10, *p* = 0.048. Inspecting the freely estimated model suggested that the paths from social skills to ODD at ages 4 and 6 might be stronger than at later ages. We thus set these two paths to be similar and the remaining paths to be similar with no change in model fit compared to the original freely estimated model, Δχ^2^ = 7.89, *df* = 9, *p* = 0.546, and this constrained model fit the data well, χ^2^ = 212.30, *df* = 123, *p* < 0.001, CFI = 0.968, TLI = 0.945, RMSEA = 0.026, 90% CI: 0.020–0.032, and was considered our final and parsimonious model (Fig. 1).

**Fig. 2:**
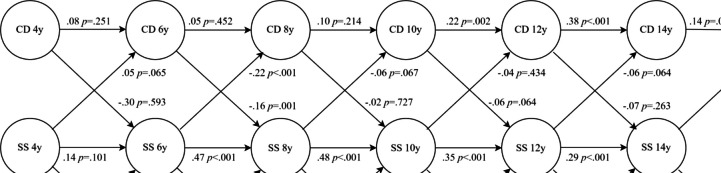
RI-CLPM showing relationships between symptoms of ODD and symptoms of CD, and teacher-reported social skills. *Note*. SS = social skills—teacher-reported; ODD = symptoms of ODD, CD = symptoms of CD. Standardized path coefficients

With regards to teacher-reported social skills, a freely estimated model did evince a sub-par fit to the data (χ^2^ = 251.84, *df* = 114, *p* < 0.001, CFI = 0.925, TLI = 0.875, RMSEA = 0.033, 90% CI: 0.028–0.039), whereas a model where 4-year autoregressions were included showed adequate fit (χ^2^ = 181.06 *df* = 100, *p* < 0.001, CFI = 0.956, TLI = 0.916, RMSEA = 0.027, 90% CI: 0.021–0.034). A model where the paths from social skills to ODD and CD were set to be equal over time did worsen the fit compared to the freely estimated model, Δχ^2^ = 35.06, *df* = 10, *p* < 0.001. However, setting the paths from social skills to ODD at ages 4–12 to be similar and letting the last cross-lagged paths vary, and setting the paths from social skills to CD to be identical across ages except for the path from age 6 to 8, did not deteriorate model fit, Δχ^2^ = 7.49, *df* = 8, *p* = 0.485. Moreover, comparing this model to a model where the paths from social skills to ODD were freely estimated (also at ages 8–12), did not change fit, Δχ^2^ = 1.73, *df* = 2, *p* = 0.227, indicating that the paths to ODD in middle childhood did not differ in strength and were stronger than the age 4 to 6, 10 to 12 and the age 14 to 16 years effects (Fig. 2). This model had favorable model fit, χ^2^ = 188.86, *df* = 108, *p* < 0.001, CFI = 0.956, TLI = 0.923, RMSEA = 0.026, 90% CI: 0.020–0.032.

### Social Skills Predicting Behavior Problems

In the above models, increased parent-rated social skills predicted reduced symptoms of CD (*β* = −0.07 to −0.15, *p* < *0.0*10) and ODD (*β* = −0.10 to −0.21, *p* < 0.001) at all time points. In the case of teacher-reported social skills, a slightly different picture emerged, where increased social skills only predicted reduced symptoms of CD at one time point, from 6 to 8 years (*β* = −0.22*, p* < 0.001). Regarding ODD, though, increased social skills predicted reduced symptoms of ODD at all time points (*β* = −0.13 to −0.14, *p* < 0.001), except for one, from age 14 to age 16 (*β* = −0.06*, p* = *0.4*65).

### Behavior Problems Predicting Social Skills

The model incorporating parent reports showed no predictive relations between increased symptoms of CD and later changes in social skills. The same held true for ODD, with the exception of one time point, from age 6 to 8 (*β* = −0.10*, p* = 0.028), but this path was rendered just beyond significance when adjusting for the false discovery rate (*p* = 0.052) Regarding teacher report, increased symptoms of CD predicted reduced social skills from age 6 to 8 years (*β* = −0.16*, p* = 0.001) only, while increased symptoms of ODD predicted reduced social skills from age 8 to 10 years (*β* = −0.12*, p* = 0.015) only.

At the between-person level, the random intercepts of ODD and CD, respectively, were not correlated with the random intercept of parent-rated social skills (*r* = 0.13, *p* = 0.628 and *r* = 0.19, *p* = 0.601). Teacher ratings correlated with ODD symptoms (*r* = −0.40, *p* = 0.001) but not with CD symptoms (*r* = −0.37, *p* = 0.097). At the within-person level, changes in CD at each time point were negatively related to changes in parent-rated social skills at that time point (*r* = −0.11, *p* = 0.041 to *r* = −19, *p* < 0.001), except at age 6 (*r* = −0.07, *p* = 0.148). Changes in ODD were consistently negatively related to parent ratings (*r* = −0.17, *p* = 0.001 to *r* = −26, *p* < 0.001). Regarding teacher ratings, only one significant relation was observed in the case of CD (age 8, *r* = −0.12, *p* = 0.041), while the rest were insignificant (*r* = −0.03, *p* = 0.663 to *r* = −08, *p* = 0.227). For ODD, a similar picture emerged, where only one relation was significant (age 8, *r* = −0.12, *p* = 0.039), while the rest were not (*r* = −0.03, *p* = 0.572 to *r* = −08, *p* = 105).

### Gender Specific Effects

Separate models were run for boys and girls, and the strengths of the paths were compared. Taking the final constrained models as vantage points, the scaled chi-square test for teacher-rated social competence revealed no gender difference (Δχ^2^ = 51.73, *df* = 42, *p* = 0.144). The model for parent-rated social skills did not converge and could not be estimated.

### CLPM Results

For the sake of comparison, we ran a traditional CLPM. All paths were set to be equal, and as in the RI-CLPM, we found that in the case of parent-rated social skills, increased social skills predicted a reduced number of both ODD- (*β* = −0.06 to −0.13, *p* > 0.001) and CD-symptoms (*β* = −0.04 to −0.07, *p* < 0.002) at all time points. Regarding the opposite direction, increased symptoms of ODD at age 6 predicting reduced social skills at age 8 (β = −0.09, p = 0.036), emerged as the only path from symptoms to later social skills. In the teacher-ratings model, the paths from 4 to 6, 12 to 14, and 14 to 16 years were set to be equal in the case of ODD, whilst for CD, all paths but those from 6 to 8 years were set to be equal. The CLPM results for teachers coincided with those of parents in terms of CD, increased social skills predicted decreased symptoms of CD at all time points (*β* = −0.04 to −0.25, *p* < *0.010*). A slightly different picture emerged for ODD, as increased social skills only predicted increased symptoms of ODD at ages 6 to 8, 8 to 10, and 10 to 12 years (*β* = −0.11 to −0.21, *p* < *0.010*). Regarding symptoms predicting social skills, increased symptoms of CD predicted reduced social skills only from age 6 to 8 years (*β* = −0.15, *p* < 0.001), whilst symptoms of ODD predicted reduced social skills from 8 to 10 years only (*β* = −0.09, *p* = 0.028). Thus, the CLPM and RI-CLPM findings for parents were similar in terms of social skills predicting later changes in disorder symptoms. For teachers, the CLPM showed a consistent relation between social skills and symptoms of CD that was not present in the RI-CLPM. Relations between social skills and ODD also differed, as the RI-CLPM showed a predictive relation from social skills to symptoms of ODD at all timepoints except the last, while the CLPM only produced the same relations in middle childhood. (Figures S2 and S3 in online supplement).

## Discussion

Poor social skills have been hypothesized to increase the risk of developing ODD and CD, but research has been limited by several methodological issues, and the findings have been mixed. We therefore examined the longitudinal reciprocal relations between social skills, as reported by parents and teachers, and DSM-5-defined symptoms of ODD and CD obtained through clinical interviews with children and parents, using a within-person methodology.

Our hypotheses were partly supported but differed with respect to respondents. Regarding parent-rated social skills, an increase in social skills predicted a reduced number of both ODD and CD symptoms at all time points. With regards to the other way around, increased symptoms of neither ODD nor CD predicted a decline in social skills. A different picture emerged with respect to teachers, as increased teacher-rated social skills only predicted reduced symptoms of CD from age 6 to 8 years. For ODD, increased social skills predicted reduced symptoms at all time points except the latest, from 14 to 16 years. In the case of increased symptoms predicting reduced social skills, increased symptoms of CD predicted reduced social skills from 6 to 8 years only, while increased symptoms of ODD predicted reduced social skills from age 8 to 10 only.

### Relations Between Social Skills and Later Symptoms of Behavior Problems

The results partly align with previous longitudinal RI-CLPM studies chronicling a predictive relation between prosocial behavior and behavioral problems (e.g., Memmott-Elison & Toseeb, 2023; Williams et al., 2024), but contrast those finding no relation (e.gSpeyer et al., 2024; Zondervan-Zwijnenburg et al., 2022) however, we go beyond this by showing that this relation is present for a wide set of social skills beyond prosociality, and for DSM-defined symptoms of ODD and (partly for) CD. Although identifying the mechanisms underlying the observed relations is outside the scope of this article, we will draw attention to some putative alternatives. First, it is conceivable that increasing social skills may lead the way for a positive developmental trajectory, which in turn leads to a downstream reduction in symptoms of ODD and CD. This dovetails with the fact that treatments focused on social skills training have led to a reduction in symptoms of ODD/CD in studies, also at follow-up (e.g., Giudice et al., 2022; van Manen et al., 2004). For example, increased socially skilled behavior may increase access to prosocial peers who will support and foster prosocial behavior. In contrast, children who have poor social skills are often rejected by socially skilled peers and are left to socialize with other aggressive children (Kendler et al., 2008; Patterson et al., 2000), who in turn may model and reinforce disruptive behavior (Patterson et al., 2000). Increased child social skills may also result in positive interactions with significant adults, perhaps leading to less conflict and harsh discipline—factors which may contribute to later reductions in symptoms of ODD and CD (e.g., Larsson et al., 2009). In that way, enhanced social skills could pave the way for continued prosocial relations as well as elicit positive responses and acceptance from prosocial others that preserve this behavior, and thereby contribute to a reduction in symptoms of ODD and CD. Thus, being accepted into prosocial groups, establishing friendships with peers, and eliciting positive responses from significant adults, could lead to a lasting positive effect that works to reduce or prevent behavior problems, evidenced by the longitudinal effects. Still, identifying such mediational effects awaits further inquiry.

We did, however, fail to see the same pattern regarding teacher-rated CD, where only one path from social skills to later symptoms emerged. One possible factor in the observed findings could be the effect of single-rater bias, as parents reported on both social skills and symptoms, which could arguably inflate the associations; something that is less likely for teachers that only reported on social skills. Furthermore, it should be noted that the social skills that parents and teachers reported on are not exactly the same, as teachers responded to the subscales cooperation, assertiveness and self-control, while parents responded to those three in addition to the subscale responsibility. The fact that we did observe a relation between teacher-rated social skills and symptoms of ODD, though, suggests that there is a difference in the social skills perceived by teachers versus those perceived by parents, specifically pertaining to the relation between social skills and later symptoms of CD. Furthermore, this difference is likely tied to situational or relational factors specific to the school or classroom environment. Social skills are context-specific, and different contexts require different skills (Gresham et al., 2011). Parents mostly view their child’s social skills as they unfold within the home, in familiar relations within the family, or with close friends. Teachers observe skills in larger group settings in a structured environment, which require participating in both hierarchical interactions with non-family adults as well as horizontal relations with a larger number of peers. It is plausible that these two situations require the use of different skills, and the teachers will likely be the ones to notice the presence or lack of these skills. With regards to this only pertaining to the social skills ➔ CD relation, the difference between ODD and CD has been getting an increasing amount of recognition, as previously mentioned. Perhaps such disorder-specific differences are reflected in these findings. Alternatively, it could be that the social skills items tap into skills that are related more so to everyday behavior difficulties that are more in line with ODD (e.g., cooperating in obeying, self-control in anger) than they are with the more serious behaviors of CD, that involve behaviors such as aggression towards others, destruction of property, deceitfulness, or serious violations of rules.

### Relations Between Symptoms of Behavior Problems and Later Social Skills

Changes in the number of symptoms of ODD and CD did not forecast later parent-rated social skills at any time point, and for only one timepoint for ODD and one for CD, with regards to teacher-rated social skills. This runs counter to findings from both some traditional cross-lagged (Burt & Roisman, 2010; Chen et al., 2010; Obradovic & Hipwell, 2010; Obsuth et al., 2015; Padilla-Walker et al., 2018) and within-person analyses (e.g., Memmott-Elison & Toseeb, 2023; Speyer et al., 2024; Williams et al., 2024; Zondervan-Zwijnenburg et al., 2022). Why our findings are at odds with other within-person results requires some scrutiny. First, despite conducting multiple statistical comparisons, none of them corrected for the false discovery rate, thus risking false positives (Benjamini & Hochberg, 2000). Secondly, the use of one single reporter on both measures may also have introduced a common-method bias in the case of Memmott-Elison and Toseeb (2023), Williams et al (2024) and Speyer et al (2024). Finally, the result may not be directly comparable to ours due to the aforementioned differences in measurements, as no previous study has specifically measured clinical symptoms of ODD and CD respectively, in conjunction with social skills. We will await further within-person studies that examine the longitudinal relations between social skills and symptoms of ODD and CD, in hopes of further elucidation of the observed findings.

In line with previous research, we did not find any gender differences in predictions (e.g., Burt & Roisman, 2010; Obsuth et al., 2015; Padilla-Walker et al., 2018) Thus, despite girls being rated more socially competent than boys across cultures, ages, and informants (Sørlie et al., 2008), whereas boys have been found to have more ODD and CD (e.g., Diamantopoulou et al., 2011), the within-person longitudinal relations do not appear to differ across gender, suggesting that reduced social skills are equally important to boys’ and girls’ development of ODD and CD.

### Limitations

Despite using a large birth cohort sample spanning 12 years and utilizing sound measures and state-of-the-art analyses that allow for the examination of within-person variance, this study has some limitations. Although we have taken steps to minimize common-method variance, parents reported on symptoms of ODD, CD and social skills, potentially inflating the longitudinal relations. Still, this was not the case for the model including teacher-reported social skills. Furthermore, findings from parent and teacher ratings were, with the exception of the relation between social skills and CD, in agreement, which is not always the case (Renk & Phares, 2004). We believe this adds confidence to the results. However, as Norwegian children generally have a lower prevalence of ODD and CD than children in many other countries (Heiervang et al., 2007), generalization to other contexts and cultures should be done with utmost care. It also needs to be mentioned that even though the RI-CLPM, by way of the random intercepts, captures all unmeasured time-invariant confounding effects, time-varying effects are not adjusted for in the same manner. However, the RI-CLPM is a very power-demanding approach, and even with only two variables included, sample sizes approaching 1,000 are typically recommended (Masselink et al., 2018). Including even more variables further reduces the power. We therefore opted not to include time-varying covariates because we would reduce the risk of reporting false negative results. However, this comes with the risk of portraying false positive effects.

## Conclusion

Increased social skills predicted later within-person reductions in DSM-5-defined symptoms of ODD and CD from preschool to middle adolescence, according to parents. Teacher-reported social skills showed the same relation to ODD from preschool to age 14, but only on one account did teacher-reported skills predict symptoms of CD. This held true for boys and girls alike. Hence, the findings support the notion that reduced social skills can be involved in the etiology of ODD and possibly also CD. They further corroborate the use of social skills training for children with symptoms of ODD and CD.

## Supplementary Information

Below is the link to the electronic supplementary material.

Supplementary file1 (DOCX 394 KB)

## References

[CR1] Achenbach, T. (1991). Manual for the child behaviour checklist/4–18 and 1991 profile. *Manual for the child behaviour checklist/4–18 and 1991 profile*. University of Vermont, Department of Psychiatry.

[CR2] Angold, A., & Costello, E. J. (2000). The Child and Adolescent Psychiatric Assessment (CAPA). *Journal of the American Academy of Child and Adolescent Psychiatry,**39*(1), 39–48. 10.1097/00004583-200001000-00015 10638066 10.1097/00004583-200001000-00015

[CR3] Azeredo, A., Moreira, D., & Barbosa, F. (2018). ADHD, CD, and ODD: Systematic review of genetic and environmental risk factors. *Research in Developmental Disabilities,**82*, 10–19. 10.1016/j.ridd.2017.12.010 29361339 10.1016/j.ridd.2017.12.010

[CR4] Beelmann, A., & Lösel, F. (2021). A comprehensive meta-analysis of randomized evaluations of the effect of child social skills training on antisocial development. *Journal of Developmental and Life-Course Criminology,**7*(1), 41–65. 10.1007/s40865-020-00142-8

[CR5] Beelmann, A., Arnold, L. S., & Hercher, J. (2023). Parent training programs for preventing and treating antisocial behavior in children and adolescents: A comprehensive meta-analysis of international studies. *Aggression and Violent Behavior,**68*, 101798. 10.1016/j.avb.2022.101798

[CR6] Benjamini, Y., & Hochberg, Y. (2000). On the adaptive control of the false discovery rate in multiple testing with independent statistics. *Journal of Educational and Behavioral Statistics,**25*(1), 60–83. 10.2307/1165312

[CR7] Bennett, K. J., & Offord, D. R. (2001). Screening for conduct problems: Does the predictive accuracy of conduct disorder symptoms improve with age? *Journal of the American Academy of Child and Adolescent Psychiatry,**40*(12), 1418–1425. 10.1097/00004583-200112000-00012 11765287 10.1097/00004583-200112000-00012

[CR8] Berry, D., & Willoughby, M. T. (2017). On the practical interpretability of cross-lagged panel models: Rethinking a developmental workhorse. *Child Development,**88*(4), 1186–1206. 10.1111/cdev.12660 27878996 10.1111/cdev.12660

[CR9] Bierman, K. L. (2004). *Peer rejection: Developmental processes and intervention strategies*. Guilford Press.

[CR10] Boldrini, T., Ghiandoni, V., Mancinelli, E., Salcuni, S., & Solmi, M. (2022). Systematic review and meta-analysis: Psychosocial treatments for disruptive behavior symptoms and disorders in adolescence. *Journal of the American Academy of Child & Adolescent Psychiatry*. 10.1016/j.jaac.2022.05.002 10.1016/j.jaac.2022.05.002 35551985

[CR11] Bornstein, M. H., Hahn, C. S., & Haynes, O. M. (2010). Social competence, externalizing, and internalizing behavioral adjustment from early childhood through early adolescence: Developmental cascades. *Development and Psychopathology,**22*(4), 717–735. 10.1017/s0954579410000416 20883577 10.1017/S0954579410000416 PMC3412561

[CR12] Bryant, F. B., & Satorra, A. (2012). Principles and practice of scaled difference chi-square testing. *Structural Equation Modeling-a Multidisciplinary Journal,**19*(3), 372–398. 10.1080/10705511.2012.687671

[CR13] Burke, J. D., Evans, S. C., & Carlson, G. A. (2022). Debate: Oppositional defiant disorder is a real disorder. *Child and Adolescent Mental Health,**27*(3), 297–299. 10.1111/camh.12588 35869580 10.1111/camh.12588

[CR14] Burke, J. D., Butler, E. J., Shaughnessy, S., Karlovich, A. R., & Evans, S. C. (2024). Evidence-based assessment of DSM-5 Disruptive, impulse control, and conduct disorders. *Assessment,**31*(1), 75–93. 10.1177/10731911231188739 37551425 10.1177/10731911231188739

[CR15] Burt, K. B., & Roisman, G. I. (2010). Competence and psychopathology: Cascade effects in the NICHD study of early child care and youth development. *Development and Psychopathology,**22*(3), 557–567. 10.1017/S0954579410000271 20576178 10.1017/S0954579410000271 PMC13152396

[CR16] Burt, K. B., Obradović, J., Long, J. D., & Masten, A. S. (2008). The interplay of social competence and psychopathology over 20 years: Testing transactional and cascade models. *Child Development,**79*(2), 359–374. 10.1111/j.1467-8624.2007.01130.x 18366428 10.1111/j.1467-8624.2007.01130.x

[CR17] Canino, G., Polanczyk, G., Bauermeister, J. J., Rohde, L. A., & Frick, P. J. (2010). Does the prevalence of CD and ODD vary across cultures? *Social Psychiatry and Psychiatric Epidemiology,**45*(7), 695–704. 10.1007/s00127-010-0242-y 20532864 10.1007/s00127-010-0242-y PMC3124845

[CR18] Chen, X. Y., Huang, X. R., Chang, L., Wang, L., & Li, D. (2010). Aggression, social competence, and academic achievement in Chinese children: A 5-year longitudinal study. *Development and Psychopathology,**22*(3), 583–592. 10.1017/s0954579410000295 20576180 10.1017/S0954579410000295

[CR19] Diamantopoulou, S., Verhulst, F. C., & van der Ende, J. (2011). The parallel development of ODD and CD symptoms from early childhood to adolescence. *European Child and Adolescent Psychiatry,**20*(6), 301–309. 10.1007/s00787-011-0175-3 21499848 10.1007/s00787-011-0175-3 PMC3098986

[CR20] Egger, H. L., Erkanli, A., Keeler, G., Potts, E., Walter, B. K., & Angold, A. (2006). Test-retest reliability of the Preschool Age Psychiatric Assessment (PAPA). *Journal of the American Academy of Child and Adolescent Psychiatry,**45*(5), 538–549. 10.1097/01.chi.0000205705.71194.b8 16601400 10.1097/01.chi.0000205705.71194.b8

[CR21] Fergusson, D. M., & Horwood, J. (1995). Predictive validity of categorically and dimensionnally scored measures of disruptive childhood behaviors. *Journal of the American Academy of Child and Adolescent Psychiatry,**34*(4), 477–485. 10.1097/00004583-199504000-00015 7751262

[CR22] Gilmour, J., Hill, B., Place, M., & Skuse, D. H. (2004). Social communication deficits in conduct disorder: A clinical and community survey. *Journal of Child Psychology and Psychiatry,**45*(5), 967–978. 10.1111/j.1469-7610.2004.t01-1-00289.x 15225339 10.1111/j.1469-7610.2004.t01-1-00289.x

[CR23] Giudice, T. D., Lindenschmidt, T., Hellmich, M., Hautmann, C., Döpfner, M., & Görtz-Dorten, A. (2022). Stability of the effects of a social competence training program for children with oppositional defiant disorder/conduct disorder: A 10-month follow-up. *European Child & Adolescent Psychiatry*. 10.1007/s00787-021-01932-1 10.1007/s00787-021-01932-1 PMC10460314 35279770

[CR24] Goertz-Dorten, A., Benesch, C., Hautmann, C., Berk-Pawlitzek, E., Faber, M., Lindenschmidt, T., Stadermann, R., Schuh, L., & Doepfner, M. (2017). Efficacy of an individualized social competence training for children with oppositional defiant disorders/conduct disorders. *Psychotherapy Research,**27*(3), 326–337. 10.1080/10503307.2015.1094587 26522864 10.1080/10503307.2015.1094587

[CR25] Goodman, R. (1997). The strengths and difficulties questionnaire: A research note. *Journal of Child Psychology and Psychiatry and Allied Disciplines,**38*(5), 581–586. 10.1111/j.1469-7610.1997.tb01545.x 9255702 10.1111/j.1469-7610.1997.tb01545.x

[CR26] Gresham, F. M., & Elliot, S. N. (1990). *Social Skills Rating System*. American Guidance Service.

[CR27] Gresham, F., & Elliott, S. N. (2008). *Social skills improvement system (SSIS) rating scales*. SSIS Rating Scales.

[CR28] Gresham, F. M., Elliott, S. N., Vance, M. J., & Cook, C. R. (2011). Comparability of the social skills rating system to the social skills improvement system: Content and psychometric comparisons across elementary and secondary age levels. *School Psychology Quarterly,**26*(1), 27–44. 10.1037/a0022662

[CR29] Hamaker, E. L., Kuiper, R. M., & Grasman, R. P. P. P. (2015). A Critique of the cross-lagged panel model. *Psychological Methods,**20*(1), 102–116. 10.1037/a0038889 25822208 10.1037/a0038889

[CR30] Hay, D. F., Hudson, K., & Liang, W. (2010). Links between preschool children’s prosocial skills and aggressive conduct problems: The contribution of ADHD symptoms. *Early Childhood Research Quarterly,**25*(4), 493–501. 10.1016/j.ecresq.2010.01.003

[CR31] Heiervang, E., Stormark, K. M., Lundervold, A. J., Heimann, M., Goodman, R., Posserud, M. B., Ullebo, A. K., Plessen, K. J., Bjelland, I., Lie, S. A., & Gillberg, C. (2007). Psychiatric disorders in Norwegian 8-to 10-year-olds: An epidemiological survey of prevalence, risk factors, and service use. *Journal of the American Academy of Child and Adolescent Psychiatry,**46*(4), 438–447. 10.1097/chi.0b013e31803062bf 17420678 10.1097/chi.0b013e31803062bf

[CR32] Hektner, J. M., August, G. J., Bloomquist, M. L., Lee, S., & Klimes-Dougan, B. (2014). A 10-year randomized controlled trial of the Early Risers conduct problems preventive intervention: Effects on externalizing and internalizing in late high school. *Journal of Consulting and Clinical Psychology,**82*(2), 355–360. 10.1037/a0035678 24447007 10.1037/a0035678

[CR33] Hendriks, A. M., Bartels, M., Colins, O. F., & Finkenauer, C. (2018). Childhood aggression: A synthesis of reviews and meta-analyses to reveal patterns and opportunities for prevention and intervention strategies. *Neuroscience and Biobehavioral Reviews,**91*, 278–291. 10.1016/j.neubiorev.2018.03.021 29580961 10.1016/j.neubiorev.2018.03.021

[CR34] Huber, L., Plotner, M., & Schmitz, J. (2019). Social competence and psychopathology in early childhood: A systematic review. *European Child & Adolescent Psychiatry,**28*(4), 443–459. 10.1007/s00787-018-1152-x 29637284 10.1007/s00787-018-1152-x

[CR35] Husby, S. M., & Wichstrøm, L. (2017). Interrelationships and continuities in symptoms of oppositional defiant and conduct disorders from age 4 to 10 in the community. *Journal of Abnormal Child Psychology,**45*(5), 947–958. 10.1007/s10802-016-0210-4 27783258 10.1007/s10802-016-0210-4 PMC5487814

[CR36] Katzmann, J., Goertz-Dorten, A., Hautmann, C., & Doepfner, M. (2019). Social skills training and play group intervention for children with oppositional-defiant disorders/conduct disorder: Mediating mechanisms in a head-to-head comparison. *Psychotherapy Research,**29*(6), 784–798. 10.1080/10503307.2018.1425559 29347904 10.1080/10503307.2018.1425559

[CR37] Kaufman, J., Birmaher, B., Brent, D., Rao, U. M. A., Flynn, C., Moreci, P., Williamson, D., & Ryan, N. (1997). Schedule for Affective Disorders and Schizophrenia for School-Age Children-Present and Lifetime Version (K-SADS-PL): Initial reliability and validity data. *Journal of the American Academy of Child & Adolescent Psychiatry,**36*(7), 980–988. 10.1097/00004583-199707000-00021 9204677 10.1097/00004583-199707000-00021

[CR38] Keenan, K., Boeldt, D., Chen, D., Coyne, C., Donald, R., Duax, J., Hart, K., Perrott, J., Strickland, J., Danis, B., Hill, C., Davis, S., Kampani, S., & Humphries, M. (2011). Predictive validity of DSM-IV oppositional defiant and conduct disorders in clinically referred preschoolers. *Journal of Child Psychology and Psychiatry,**52*(1), 47–55. 10.1111/j.1469-7610.2010.02290.x 20738448 10.1111/j.1469-7610.2010.02290.x PMC3005994

[CR39] Kendler, K. S., Jacobson, K., Myers, J. M., & Eaves, L. J. (2008). A genetically informative developmental study of the relationship between conduct disorder and peer deviance in males. *Psychological Medicine,**38*(7), 1001–1011. 10.1017/S0033291707001821 17935643 10.1017/S0033291707001821 PMC4248600

[CR40] Kolko, D. J., Dorn, L. D., Bukstein, O. G., Pardini, D., Holden, E. A., & Hart, J. (2009). Community vs. clinic-based modular treatment of children with early-onset ODD or CD: A clinical trial with 3-year follow-up. *Journal of Abnormal Child Psychology,**37*(5), 591–609. 10.1007/s10802-009-9303-7 19221871 10.1007/s10802-009-9303-7 PMC4986609

[CR41] Larsson, B., Fossum, S., Clifford, G., Drugli, M. B., Handegard, B. H., & Morch, W.-T. (2009). Treatment of oppositional defiant and conduct problems in young Norwegian children results of a randomized controlled trial. *European Child & Adolescent Psychiatry,**18*(1), 42–52. 10.1007/s00787-008-0702-z 18563473 10.1007/s00787-008-0702-z

[CR42] Li, C. (2013). Little’s test of missing completely at random. *Stata Journal,**13*(4), 795–809. 10.1177/1536867x1301300407

[CR43] Little, S. G., Swangler, J., & Akin-Little, A. (2017). Defining Social Skills. In J. L. Matson (Ed.), *Handbook of Social Behavior and Skills in Children* (pp. 9–17). Springer International Publishing. 10.1007/978-3-319-64592-6_2

[CR44] Loeber, R., Green, S. M., Lahey, B. B., Frick, P. J., & McBurnett, K. (2000). Findings on disruptive behavior disorders from the first decade of the developmental trends study. *Clinical Child and Family Psychology Review,**3*(1), 37–60. 10.1023/a:1009567419190 11228766 10.1023/a:1009567419190

[CR45] Masselink, M., Van Roekel, E., Hankin, B. L., Keijsers, L., Lodder, G. M. A., Vanhalst, J., Verhagen, M., Young, J. F., & Oldehinkel, A. J. (2018). The longitudinal association between self-esteem and depressive symptoms in adolescents: Separating between-person effects from within-person effects. *European Journal of Personality,**32*(6), 653–671. 10.1002/per.2179 31105382 10.1002/per.2179 PMC6519152

[CR46] Memmott-Elison, M. K., & Toseeb, U. (2023). Prosocial behavior and psychopathology: An 11-year longitudinal study of inter- and intraindividual reciprocal relations across childhood and adolescence. *Development and Psychopathology,**35*(4), 1982–1996. 10.1017/S0954579422000657 35957579 10.1017/S0954579422000657

[CR47] Merrell, K. W. (2001). Assessment of children’s social skills: Recent developments, best practices, and new directions. *Exceptionality,**9*(1–2), 3–18.

[CR48] Obradovic, J., & Hipwell, A. (2010). Psychopathology and social competence during the transition to adolescence: The role of family adversity and pubertal development. *Development and Psychopathology,**22*(3), 621–634. 10.1017/s0954579410000325 20576183 10.1017/S0954579410000325 PMC2892816

[CR49] Obsuth, I., Eisner, M. P., Malti, T., & Ribeaud, D. (2015). The developmental relation between aggressive behaviour and prosocial behaviour: A 5-year longitudinal study. *BMC Psychology,**3*(1), 16. 10.1186/s40359-015-0073-4 26000166 10.1186/s40359-015-0073-4 PMC4440499

[CR50] Padilla-Walker, L. M., Memmott-Elison, M. K., & Coyne, S. M. (2018). Associations between prosocial and problem behavior from early to late adolescence. *Journal of Youth and Adolescence,**47*(5), 961–975. 10.1007/s10964-017-0736-y 28866855 10.1007/s10964-017-0736-y

[CR51] Patterson, G. R., Dishion, T. J., & Yoerger, K. (2000). Adolescent growth in new forms of problem behavior: Macro- and micro-peer dynamics. *Prevention Science,**1*(1), 3–13. 10.1023/a:1010019915400 11507792 10.1023/a:1010019915400

[CR52] Renk, K., & Phares, V. (2004). Cross-informant ratings of social competence in children and adolescents. *Clinical Psychology Review,**24*(2), 239–254. 10.1016/j.cpr.2004.01.004 15081518 10.1016/j.cpr.2004.01.004

[CR53] Riise, E. N., Wergeland, G. J. H., Njardvik, U., & Öst, L.-G. (2021). Cognitive behavior therapy for externalizing disorders in children and adolescents in routine clinical care: A systematic review and meta-analysis. *Clinical Psychology Review,**83*, 101954. 10.1016/j.cpr.2020.101954 33418192 10.1016/j.cpr.2020.101954

[CR54] Rolon-Arroyo, B., Arnold, D. H., & Harvey, E. A. (2014). The predictive utility of conduct disorder symptoms in preschool children: A 3-year follow-up study. *Child Psychiatry and Human Development,**45*(3), 329–337. 10.1007/s10578-013-0404-8 23979222 10.1007/s10578-013-0404-8 PMC4707669

[CR55] Rose-Krasnor, L. (1997). The nature of social competence: A theoretical review. *Social Development,**6*(1), 111–135. 10.1111/j.1467-9507.1997.tb00097.x

[CR56] Sørlie, M.-A., Hagen, K. A., & Ogden, T. (2008). Social competence and antisocial behavior: Continuity and distinctiveness across early adolescence. *Journal of Research on Adolescence,**18*(1), 121–144. 10.1111/j.1532-7795.2008.00553.x

[CR57] Speyer, L. G., Obsuth, I., Eisner, M., Ribeaud, D., & Murray, A. L. (2024). Does prosociality in early-to mid-adolescence protect against later development of antisocial behaviours? *The Journal of Early Adolescence,**44*(9), 1124–1153. 10.1177/02724316231210254 39372428 10.1177/02724316231210254 PMC11446672

[CR58] Steinsbekk, S., & Wichstrom, L. (2018). Cohort Profile: The Trondheim Early Secure Study (TESS)-a study of mental health, psychosocial development and health behaviour from preschool to adolescence. *International Journal of Epidemiology*. 10.1093/ije/dyy190 30215720 10.1093/ije/dyy190

[CR59] Sveen, T. H., Berg-Nielsen, T. S., Lydersen, S., & Wichstrøm, L. (2013). Detecting psychiatric disorders in preschoolers: Screening with the strengths and difficulties questionnaire. *Journal of the American Academy of Child & Adolescent Psychiatry,**52*(7), 728–736. 10.1016/j.jaac.2013.04.010 23800486 10.1016/j.jaac.2013.04.010

[CR60] van Manen, T. G., Prins, P. J. M., & Emmelkamp, P. M. G. (2004). Reducing aggressive behavior in boys with a social cognitive group treatment: Results of a randomized, controlled trial. *Journal of the American Academy of Child & Adolescent Psychiatry,**43*(12), 1478–1487. 10.1097/01.chi.0000142669.36815.3e 15564817 10.1097/01.chi.0000142669.36815.3e

[CR61] Webster-Stratton, C., & Reid, M. J. (2018). The Incredible Years parents, teachers, and children training series: A multifaceted treatment approach for young children with conduct problems. In *Evidence-based psychotherapies for children and adolescents, 3rd ed.* (pp. 122–141). The Guilford Press.

[CR62] Wichstrøm, L., Belsky, J., Jozefiak, T., Sourander, A., & Berg-Nielsen, T. S. (2014). Predicting service use for mental health problems among young children. *Pediatrics,**133*(6), 1054–1060. 10.1542/peds.2013-3184 24819574 10.1542/peds.2013-3184

[CR63] Wiesner, M., Elliott, M. N., McLaughlin, K. A., Banspach, S. W., Tortolero, S., & Schuster, M. A. (2015). Common versus specific correlates of fifth-grade conduct disorder and oppositional defiant disorder symptoms: Comparison of three racial/ethnic groups. *Journal of Abnormal Child Psychology,**43*(5), 985–998. 10.1007/s10802-014-9955-9 25411125 10.1007/s10802-014-9955-9 PMC4439379

[CR64] Williams, C., McGee, T. R., Walding, S., & Bond, C. E. W. (2024). The role of prosocial behaviour in the deceleration of conduct problem behaviour. *Journal of Developmental and Life-Course Criminology*. 10.1007/s40865-024-00256-3

[CR65] Zhang, W. R., He, T., Hinshaw, S., Chi, P. L., & Lin, X. Y. (2023). Longitudinal relationship between oppositional defiant disorder symptoms and attention-deficit/hyperactivity disorder symptoms in Chinese children: Insights from cross-lagged panel network analyses. *European Child & Adolescent Psychiatry*. 10.1007/s00787-023-02347-w 10.1007/s00787-023-02347-w 38151686

[CR66] Zondervan-Zwijnenburg, M., Dobbelaar, S., van der Meulen, M., & Achterberg, M. (2022). Longitudinal associations between prosocial behavior and behavioral problems across childhood: A robust random-intercept cross-lagged panel model. *Developmental Psychology,**58*(6), 1139–1155. 10.1037/dev0001346 35446069 10.1037/dev0001346

