# Supplementary material for: Within-person Relations Between Social Skills and Symptoms of Oppositional Defiant Disorder and Conduct Disorder from Preschool to Adolescence – A Birth Cohort Study
Source: Res Child Adolesc Psychopathol. 2025 Feb 18;53(4):473–84. doi: 10.1007/s10802-025-01298-x (PMC12031923; doi:10.1007/s10802-025-01298-x)
Supplement: Supplementary file 1 — Supplementary file1 (DOCX 394 KB) [file 10802_2025_1298_MOESM1_ESM.docx]

Table S1

*Means and SD of the study variables*

| Variable | Means | SD |
| --- | --- | --- |
| SST4y | 2.88 | .40 |
| SST6y | 2.88 | .43 |
| SST8y | 2.97 | .46 |
| SST10y | 2.92 | .46 |
| SST12y | 3.03 | .44 |
| SST14y | 2.94 | .45 |
| SSP4y | 2.59 | .26 |
| SSP6y | 2.78 | .31 |
| SSP8y | 2.87 | .36 |
| SSP10y | 2.94 | .35 |
| SSP12y | 3.24 | .35 |
| SSP14y | 3.24 | .33 |
| SSP16y | 3.18 | .32 |
| ODD4y | .89 | 1.25 |
| ODD6y | 1.18 | 1.34 |
| ODD8y | 1.84 | 1.52 |
| ODD10y | 1.39 | 1.62 |
| ODD14y | 1.07 | 1.39 |
| ODD16y | .20 | .65 |
| CD4y | .41 | .70 |
| CD6y | .29 | .54 |
| CD8y | .34 | .65 |
| CD10y | .28 | .62 |
| CD12y | .26 | .59 |
| CD14y | .27 | .61 |
| CD16y | .51 | 1.23 |

*Note: SST=social skills – teacher-reported; SSP=social skills – parent-reported; ODD=symptoms of ODD; CD=symptoms of CD. Raw scores, not log transforme*

Table S2

*Correlations between the study variables*

|  | SST4 | SST6 | SST8 | SST10 | SST12 | SST14 | SSP4 | SSP6 | SSP8 | SSP10 | SSP12 | SSP14 | SSP16 | ODD4 | ODD6 | ODD8 | ODD10 | ODD12 | ODD14 | ODD16 | CD4 | CD6 | CD8 | CD10 | CD12 | CD14 | CD16 |
| --- | --- | --- | --- | --- | --- | --- | --- | --- | --- | --- | --- | --- | --- | --- | --- | --- | --- | --- | --- | --- | --- | --- | --- | --- | --- | --- | --- |
| SST4 | - |  |  |  |  |  |  |  |  |  |  |  |  |  |  |  |  |  |  |  |  |  |  |  |  |  |  |
| SST6 | .31^c^ | - |  |  |  |  |  |  |  |  |  |  |  |  |  |  |  |  |  |  |  |  |  |  |  |  |  |
| SST8 | .32^c^ | .59 | - |  |  |  |  |  |  |  |  |  |  |  |  |  |  |  |  |  |  |  |  |  |  |  |  |
| SST10 | .31^c^ | .47^c^ | .64^c^ | - |  |  |  |  |  |  |  |  |  |  |  |  |  |  |  |  |  |  |  |  |  |  |  |
| SST12 | .28 | .34^c^ | .50^c^ | .56^c^ | - |  |  |  |  |  |  |  |  |  |  |  |  |  |  |  |  |  |  |  |  |  |  |
| SST14 | .21^c^ | .36^c^ | .40^c^ | .34^c^ | .44^c^ | - |  |  |  |  |  |  |  |  |  |  |  |  |  |  |  |  |  |  |  |  |  |
| SSP4 | .17^c^ | .18^c^ | .13^a^ | .12^a^ | .08n | .12^n^ | - |  |  |  |  |  |  |  |  |  |  |  |  |  |  |  |  |  |  |  |  |
| SSP6 | .18^c^ | .26^c^ | .22^c^ | .19^c^ | .18^c^ | .12^n^ | .61^c^ | - |  |  |  |  |  |  |  |  |  |  |  |  |  |  |  |  |  |  |  |
| SSP8 | .14^b^ | .24^c^ | .22^c^ | .22^c^ | .21^c^ | .13^a^ | .50^c^ | .70^c^ | - |  |  |  |  |  |  |  |  |  |  |  |  |  |  |  |  |  |  |
| SSP10 | .15^b^ | .23^c^ | .29^c^ | .34^c^ | .26^c^ | .12^c^ | .48^c^ | .67^c^ | .74^c^ | - |  |  |  |  |  |  |  |  |  |  |  |  |  |  |  |  |  |
| SSP12 | .12^c^ | .22^c^ | .30^c^ | .34^c^ | .23^c^ | .23^c^ | .30c | .51^c^ | .56^c^ | .69^c^ | - |  |  |  |  |  |  |  |  |  |  |  |  |  |  |  |  |
| SSP14 | .15^b^ | .24^c^ | .26^c^ | .28^c^ | .29^c^ | .29^c^ | .39^c^ | .51^c^ | .53^c^ | .65^c^ | .71^c^ | - |  |  |  |  |  |  |  |  |  |  |  |  |  |  |  |
| SSP16 | .06^n^ | .16^c^ | .18^b^ | .25^c^ | .26^c^ | .22^c^ | .31^c^ | .37^c^ | .43^c^ | .54^c^ | .55^c^ | .60^c^ | - |  |  |  |  |  |  |  |  |  |  |  |  |  |  |
| ODD4 | -.07^n^ | -.08^n^ | -.09^n^ | -.04^n^ | -.04^n^ | -.11^a^ | -.21^c^ | -.15^c^ | -.11^a^ | -.15^b^ | -.23^c^ | -.08^n^ | -.07^n^ | - |  |  |  |  |  |  |  |  |  |  |  |  |  |
| ODD6 | -.12^a^ | -.10^a^ | -.16^b^ | -.16^b^ | -.16^b^ | -.12^a^ | -.16^c^ | -.25^c^ | -.24^c^ | -.22^c^ | -.27^c^ | -.19^c^ | -.14^b^ | .28^c^ | - |  |  |  |  |  |  |  |  |  |  |  |  |
| ODD8 | -.20^c^ | -.26^c^ | -.29^c^ | -.29^c^ | -.24^c^ | -.15^a^ | -.24^c^ | -.29^c^ | -.32^c^ | -.30^c^ | -.32^c^ | -.26^c^ | -.20^c^ | .31^c^ | .37^c^ | - |  |  |  |  |  |  |  |  |  |  |  |
| ODD10 | -.23^c^ | -.21^c^ | -.33^c^ | -.33^c^ | -.23^c^ | -.19^b^ | -.17^c^ | -.23^c^ | -.22^c^ | -.32^c^ | -.29^c^ | -.28^c^ | -.28^c^ | .31^c^ | .36^c^ | .49^c^ | - |  |  |  |  |  |  |  |  |  |  |
| ODD12 | -.20^c^ | -.18^c^ | -.35^c^ | -.35^c^ | -.27^c^ | -.21^c^ | -.11^a^ | -.21^c^ | -.25^c^ | -.28^c^ | -.34^c^ | -.27^c^ | -.21^c^ | .26^c^ | .33^c^ | .42^c^ | .48^c^ | - |  |  |  |  |  |  |  |  |  |
| ODD14 | -.14^b^ | -.09^n^ | -.28^c^ | -.28^c^ | -.23^c^ | -.20^c^ | -.05^n^ | -.17^b^ | -.19^c^ | -.29^c^ | -.31^c^ | -.35^c^ | -.23^c^ | .22^c^ | .24^c^ | .32^c^ | .42^c^ | .44^c^ | - |  |  |  |  |  |  |  |  |
| ODD16 | -.09^n^ | -.14^b^ | -.18^b^ | -.18^b^ | -.23^c^ | -.11^n^ | -.02^n^ | -.09^n^ | -.10^a^ | -.15^b^ | -.17^b^ | -.15^b^ | -.22^c^ | .14^b^ | .23^c^ | .31^c^ | .32^c^ | .29^c^ | .27^c^ | - |  |  |  |  |  |  |  |
| CD4 | -.07^n^ | -.07^n^ | -.04^n^ | -.04^n^ | -.01^n^ | -.08^n^ | -.11^b^ | -.09^a^ | -.04^n^ | -.05^n^ | -.13^a^ | -.04^n^ | -.02^n^ | .32^c^ | .14^b^ | .15^b^ | .20^c^ | .08^n^ | .21^c^ | -.03^n^ | - |  |  |  |  |  |  |
| CD6 | -.04^n^ | -.08^n^ | -.22^c^ | -.22^c^ | -.11^a^ | -.13^a^ | -.09^a^ | -.11^a^ | -.09^a^ | -.09^n^ | -.12^a^ | -.12^a^ | -.10^n^ | .24^c^ | .32^c^ | .19^c^ | .17^c^ | .12^a^ | .10^n^ | .07^n^ | .20^c^ | - |  |  |  |  |  |
| CD8 | -.12^a^ | -.27^c^ | -.28^c^ | -.28^c^ | -.21^c^ | -.16^b^ | -.06^a^ | -.13^b^ | -.18^c^ | -.18^c^ | -.22^c^ | -.18^c^ | -.17^b^ | .09^n^ | .19^c^ | .42^c^ | .22^c^ | .15^b^ | .21^c^ | .17^b^ | .10^a^ | .16^b^ | - |  |  |  |  |
| CD10 | -.02^n^ | -.15^b^ | -.17^b^ | -.17^b^ | -.15^b^ | -.03^n^ | -.04^a^ | -.11^a^ | -.16^c^ | -.23^c^ | -.23^c^ | -.15^b^ | -.19^c^ | .10^a^ | .19^c^ | .25^c^ | .34^c^ | .24^c^ | .25^c^ | .15^a^ | .17^b^ | .14^b^ | .24^c^ | - |  |  |  |
| CD12 | -.17^b^ | -.13^b^ | -.25^b^ | -.25^c^ | -.21^c^ | -.20^c^ | -.05^a^ | -.13b | -.14^b^ | -.20^c^ | -.28^c^ | -.24^c^ | -.16^b^ | .08^n^ | .21^c^ | .24^c^ | .27^c^ | .36^c^ | .35^c^ | .13^n^ | .22^c^ | .18b | .27^c^ | .35^c^ | - |  |  |
| CD14 | -.10^a^ | -.10^n^ | -.16^b^ | -.16^b^ | -.17^b^ | -.17^b^ | -.05^a^ | -.05^n^ | -.08^n^ | -.23^c^ | -.20^c^ | -.27^c^ | -.20^c^ | .05^n^ | .14^b^ | .19^b^ | .31^c^ | .23^c^ | .31^c^ | .24^c^ | .09^n^ | .13^a^ | .18^b^ | .37^c^ | .50^c^ | - |  |
| CD16 | -.12^a^ | -.08^n^ | -.19^b^ | -.19^b^ | -.23^c^ | -.16^b^ | -.06^a^ | -.15^c^ | -.16^c^ | -.18^b^ | -.24^c^ | -.18^b^ | -.22^c^ | .08^n^ | .18^b^ | .24^c^ | .29^c^ | .30^c^ | .29^c^ | .54^c^ | .01^n^ | .04^n^ | .17^b^ | .14^a^ | .17^a^ | .24^c^ | - |

*Note: SST=social skills – teacher-reported; SSP=social skills – parent-reported; ODD=symptoms*

*of ODD; CD=symptoms of CD*. ^a^ p < 0.05, ^b^ p < 0.01, ^c^ p < 0.001, ^n^ = non-significant

Figure S2

CLPM of relations between symptoms of ODD, symptoms of CD, and parent-reported social skills.
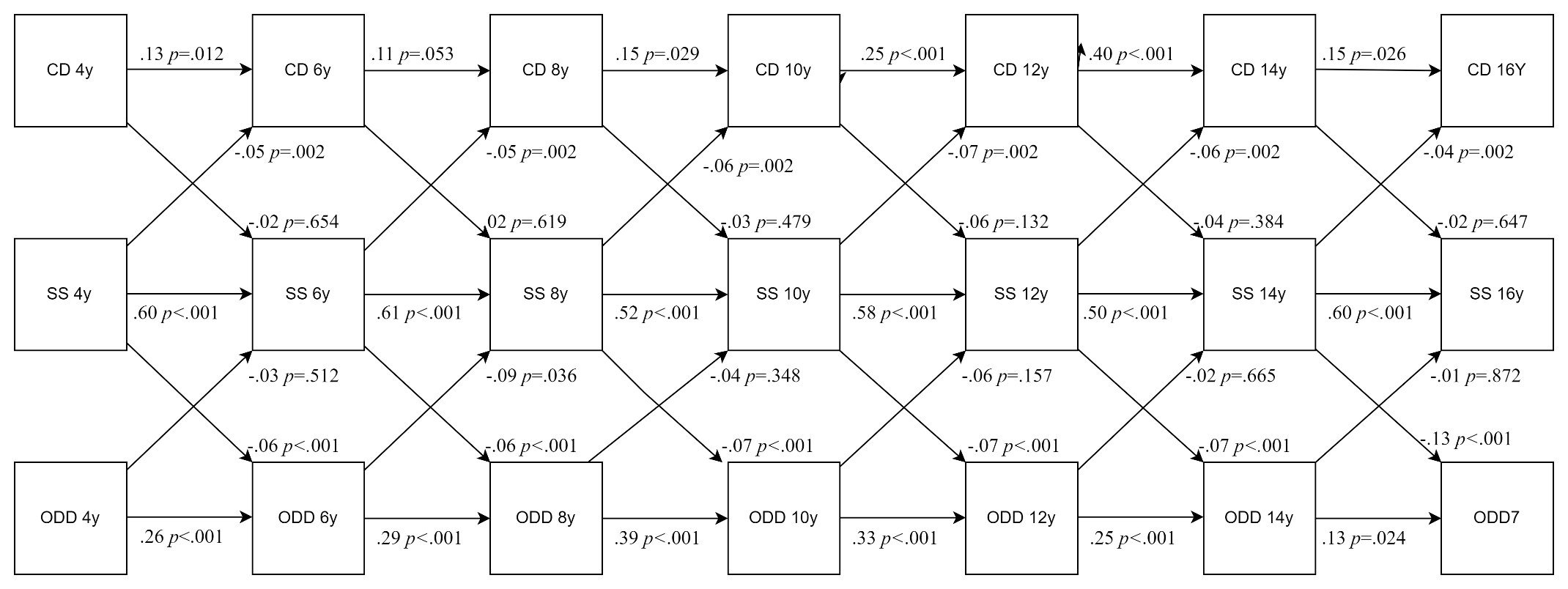


*Note*. SS=social skills - parent-reported; ODD=symptoms of ODD, CD=symptoms of CD. Standardized path coefficients.

Figure S3

CLPM of relations between symptoms of ODD, symptoms of CD, and teacher-reported social skills.


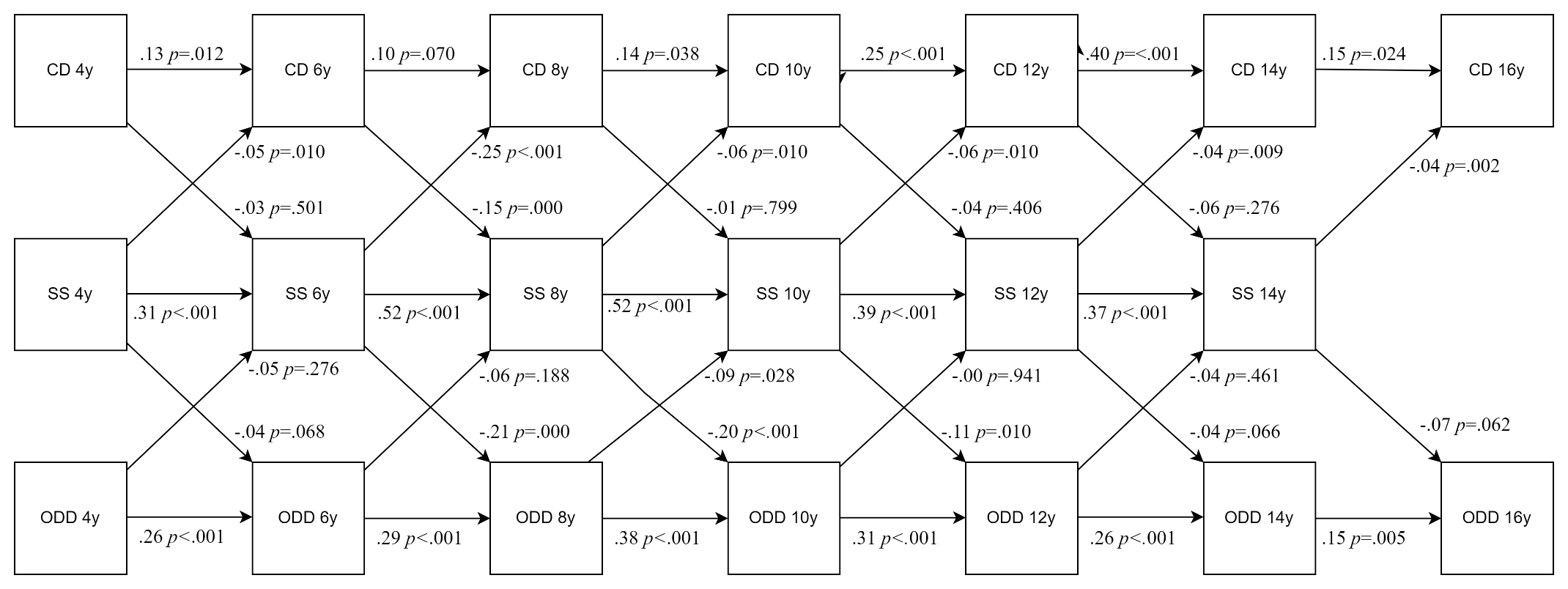
*Note*. SS=social skills - teacher-reported; ODD=symptoms of ODD, CD=symptoms of CD. Standardized path coefficients.
